# Fabrication of 2-D Capacitive Micromachined Ultrasonic Transducer (CMUT) Array through Silicon Wafer Bonding

**DOI:** 10.3390/mi13010099

**Published:** 2022-01-08

**Authors:** Ziyuan Wang, Changde He, Wendong Zhang, Yifan Li, Pengfei Gao, Yanan Meng, Guojun Zhang, Yuhua Yang, Renxin Wang, Jiangong Cui, Hongliang Wang, Binzhen Zhang, Yongfeng Ren, Guoyong Zhen, Xinquan Jiao, Sai Zhang

**Affiliations:** State Key Laboratory of Dynamic Testing Technology, North University of China, Taiyuan 030051, China; ziyuanwang1996@163.com (Z.W.); hechangde@nuc.edu.cn (C.H.); 18234102861@163.com (Y.L.); gaopengfei988@163.com (P.G.); myn18035690408@163.com (Y.M.); Zhangguojun1977@nuc.edu.cn (G.Z.); yangyuhua407@163.com (Y.Y.); wangrenxin@pku.edu.cn (R.W.); jgcui@nuc.edu.cn (J.C.); wanghongliang@nuc.edu.cn (H.W.); zhangbinzhen@nuc.edu.cn (B.Z.); renyongfeng@nuc.edu.cn (Y.R.); zengguoyong@nuc.edu.cn (G.Z.); jiaoxinquan@nuc.edu.cn (X.J.); zhangsai@ujs.edu.cn (S.Z.)

**Keywords:** MEMS sensors, CMUT array, FEM, high-sensitivity, broadband

## Abstract

Capacitive micromachined ultrasound transducers (CMUTs) have broad application prospects in medical imaging, flow monitoring, and nondestructive testing. CMUT arrays are limited by their fabrication process, which seriously restricts their further development and application. In this paper, a vacuum-sealed device for medical applications is introduced, which has the advantages of simple manufacturing process, no static friction, repeatability, and high reliability. The CMUT array suitable for medical imaging frequency band was fabricated by a silicon wafer bonding technology, and the adjacent array devices were isolated by an isolation slot, which was cut through the silicon film. The CMUT device fabricated following this process is a 4 × 16 array with a single element size of 1 mm × 1 mm. Device performance tests were conducted, where the center frequency of the transducer was 3.8 MHz, and the 6 dB fractional bandwidth was 110%. The static capacitance (29.4 pF) and center frequency (3.78 MHz) of each element of the array were tested, and the results revealed that the array has good consistency. Moreover, the transmitting and receiving performance of the transducer was evaluated by acoustic tests, and the receiving sensitivity was −211 dB @ 3 MHz, −213 dB @ 4 MHz. Finally, reflection imaging was performed using the array, which provides certain technical support for the research of two-dimensional CMUT arrays in the field of 3D ultrasound imaging.

## 1. Introduction

Ultrasonic technology is widely used in medical diagnosis, underwater detection, and industrial nondestructive testing [1]. Ultrasonic imaging is based on the reflection, refraction, and transmission of sound propagating in different media [2,3,4,5,6,7]. Ultrasonic transducer is the key device in ultrasonic applications, and is used to transmit and receive ultrasonic waves. With the development of micro-electro-mechanical systems (MEMS) [8], MEMS-based ultrasonic transducers have attracted increasing attention. Currently, piezoelectric ultrasonic transducers still occupy a dominant position in ultrasonic applications [1]. However, their acoustic impedance is significantly higher than that of fluid media and air, which significantly limits their performance [9]. To overcome this problem, it is necessary to make corresponding matching layers, which will be used to eliminate excessive reflections on the interface of the two media due to the excessive difference in acoustic impedance [10]. The emergence of capacitive micromachined ultrasonic transducers (CMUTs) has solved the problem related to the acoustic impedance. The emission source of CMUTs is a very thin film; thus, the acoustic impedance is low and matches better that of the fluid medium [11]. In addition, CMUTs have the advantages of large immersion bandwidth, high directivity, wide working temperature range, low-cost manufacturing, and easy integration with the integrated circuit (IC), making them strong candidates for ultrasonic applications [12,13,14,15]. The advantages of easy array of CMUT also make three-dimensional ultrasonic imaging gradually realized. Three-dimensional ultrasonic imaging can be realized by controlling the CMUT array through phased array.

At present, CMUT arrays can be successfully prepared in various ways. Zhang et al. (2019) designed a CMUT one-dimensional (1D) linear array (1 × 72 elements) for underwater long-range imaging [16]. Adelegan et al. (2019) prepared a CMUT two-dimensional (2D) array following a sacrificial release process, and successfully tested its performance [17]. In 2015, Lavanderamucia et al. (2015) used anodic bonding to prepare 2D arrays [18]. Devices prepared by wafer bonding have more advantages in producing a closed cavity structure, and have better performance in the control of cavity size and shape, as well as membrane material uniformity [2,19,20].

In this study, considering the performance, bandwidth, and sensitivity of the transducer array, the transducer array was fabricated using MEMS processing technology. More specifically, a transducer with a thickness of 2 μm and a center frequency of 3 MHz was prepared by silicon wafer bonding, which is suitable for underwater ultrasonic imaging [21]. The vibration film is made of monocrystalline silicon, and its stability and reliability were verified. Currently, the interconnection of the 2D CMUT array is based on the through silicon via (TSV) [22]. In this technology, lithography etching of the positive and negative sides needs to be repeatedly performed, and the depth–width ratio and verticality of the through hole are higher, which increases the preparation steps and undoubtedly increases the preparation difficulty. In this study, the Ag–Al wire bonding method was used for interconnecting the array of transducers [23]. This interconnection method has been proved to have strong stability, the introduction of silicon pores can be easily avoided, and the preparation steps are simplified. In addition, the array introduces an isolation slot to isolate each element, preventing interference between elements. Finally, the acoustic characteristics and the device characteristics of the transducer were assessed.

## 2. Materials and Methods

### 2.1. Transducer Structure

**Figure 1:**
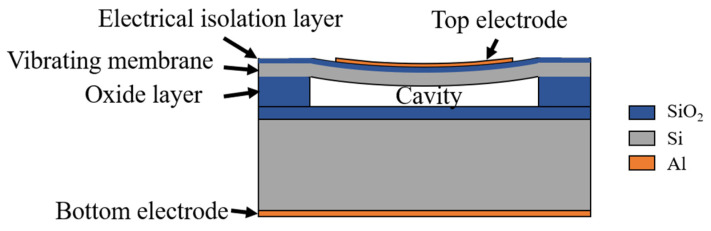
Cross-sectional view and components of a single cell of the proposed CMUT.

Each element in the CMUT is composed of multiple cells arranged in a 1D or 2D structure. Each cell is a parallel capacitor plate structure composed of electrodes, diaphragms, and cavities. The metal aluminum electrode and the silicon oxide layer are sequentially fixed on the surface of the diaphragm, and the silicon oxide acts as an electrical isolation. The vibration film is formed by the device layer of the silicon-on-insulator (SOI) wafer. The dielectric layer of the capacitance structure is formed by the vacuum cavity etched by the silicon oxide layer. The bottom part is composed of the silicon substrate layer and the metal aluminum electrode attached to the bottom surface. A schematic illustration of the CMUT cell structure is shown in Figure 1. When the CMUT is working, a DC bias voltage is often applied to make the film sink into the cavity; this way, the film stress increases the sensitivity of the transducer. When transmitting ultrasound, the AC signal should be superimposed on the applied DC bias voltage, and the driving frequency of the AC signal is the transmission frequency of ultrasound. When receiving, the conversion of mechanical energy into electrical signals is performed through the diaphragm. Thus far, the transducer structures of CMUTs applied in medical diagnosis, underwater detection, and industrial nondestructive testing are basically the same [24].

In order to design the working frequency of the transducer at 3–4 MHz and ensure that the ultrasonic frequency has high resolution and appropriate penetration, the CMUT structure was analyzed by the COMSOL finite element analysis (FEA) software and the size was obtained based on theoretical calculations. The resonant frequency of the CMUT structure can be expressed as Equation (1). The resonant frequency of the transducer was 3.8 MHz, and the dimensions of the CMUT structure are listed in Table 1. The transducer is designed for liquid environment and provides transducer support for future underwater three-dimensional (3D) ultrasound imaging. Each element in the transducer is composed of multiple cells, which can improve the ultrasound transmitting and receiving ability. Moreover, isolation grooves between each element are also prepared to avoid the potential interference between elements. The silicon oxide electrical isolation layer is inserted between the upper electrode and the silicon vibration film to prevent capacitance coupling.

(1) $$f_{0}=\frac{0.47t_{m}}{a^{2}}\sqrt{\frac{E}{\rho \left(1-\sigma^{2}\right)}},$$

*E* is the Young’s modulus, ρ is the density of the membrane material, σ is the Poisson’s ratio, a is the radius of the circular membrane, and $t_{m}$ is the thickness of the membrane.

**Figure 2:**
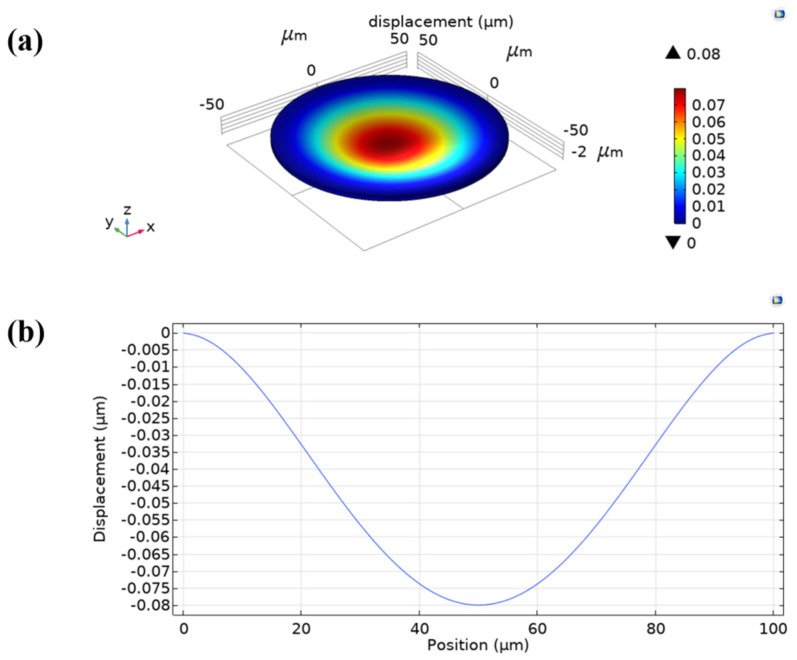
Membrane displacement under the applied atmospheric pressure. (**a**) 3D membrane displacement contour. (**b**) Displacement vs. position graph.

**Figure 3:**
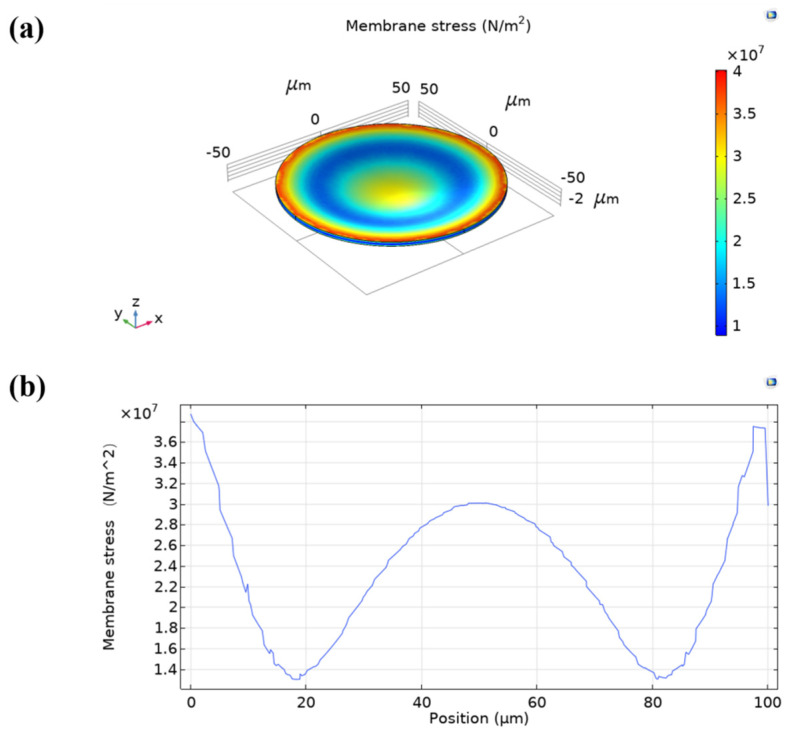
Membrane stress under the applied atmospheric pressure. (**a**) 3D stress contour. (**b**) Stress vs. position graph.

A 3D model of the same scale was developed in the FEA software. In the simulation, a fixed constraint was applied around the silicon vibration film and the silicon substrate, an atmospheric pressure (101 kPa) was applied on the model surface, and the displacement of the membrane under atmospheric pressure was 0.08 µm (Figure 2). The stress in the membrane was 38 MPa, which was less than 100 MPa (Figure 3). When the silicon vibration film is vibrating, the internal stress changes, and the fracture strength of silicon is approximately 80–140 MPa [25]. In general, a single CMUT cannot meet the directivity, acoustic power, and information processing requirements in practical applications. The final element size is determined by the directivity of the array, and the proper spacing of the array elements makes the emitted sound waves have no shales. The number of cells is limited for determining the frequency structure and spacing of the array elements. For increasing the driving voltage to increase the transmitting sound pressure is limited by the stress of the film, the applied voltage cannot exceed the collapse voltage. Therefore, within a fixed area, the number of capacitive structures of the same silicon diaphragm material at a specific frequency is basically determined. In this structure, 35 cells are selected. Thus, the CMUT is arranged in a certain way to combine the array.

### 2.2. CMUT Array Fabrication

**Figure 4:**
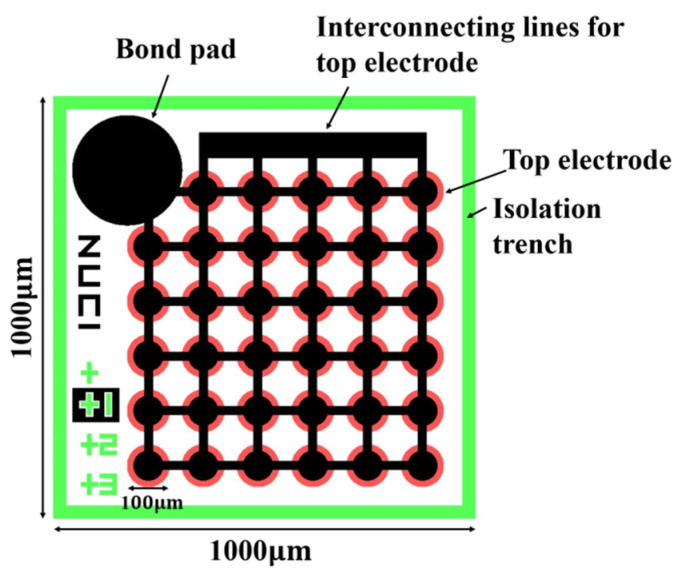
CMUT composite mask layout (35 cells).

**Figure 5:**
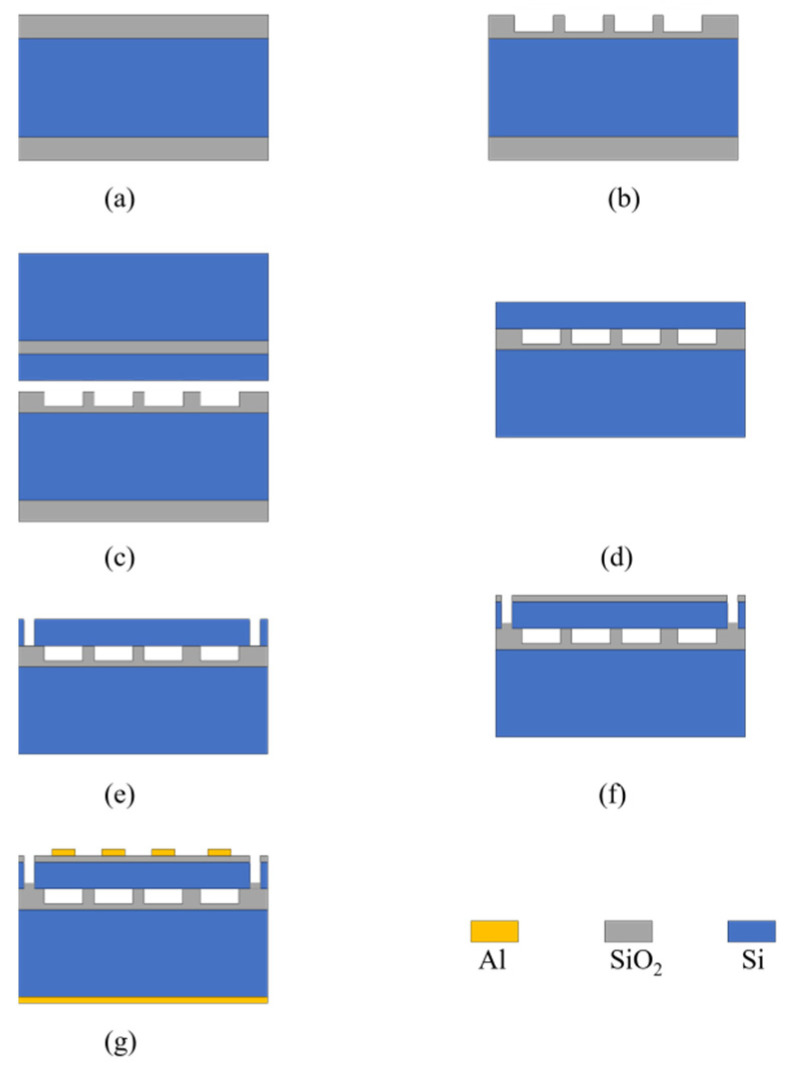
Process flow diagram: (**a**) Oxide wafer and SOI wafer preparation; (**b**) Etching of the cavity on the oxide layer of the oxide sheet; (**c**) Silicon wafer bonding of oxide wafers and SOI wafers; (**d**) Substrate layer and Buried Oxide Layer thinning; (**e**) Etching of the isolation slot on the membrane; (**f**) Silicon oxide deposition as an electrical isolation layer; (**g**) The metal is deposited and patterned as the upper electrode, and the entire lower surface is sputtered as the lower electrode.

After the design and analysis steps, the photolithography mask was designed in L-Edit. Subsequently, a three-layer mask was prepared, which also reflected the simplicity of the preparation steps. The CMUT preparation process requires only three masks, and the main difficulty is the silicon wafer bonding process. The overlay effect of the mask is exhibited in Figure 4. In the preliminary preparation phase, two wafers are needed; the silicon wafer on the low resistivity silicon substrate, and the SOI wafer with a device layer thickness of 2 μm, which was prepared according to the process described in detail in the flow diagram in Figure 5.

#### 2.2.1. Wafer Preparation

Firstly, a 4-inch oxide wafer was prepared, and the resistivity of the silicon substrate was 0.0015 Ω·cm, which provided good conductivity with the lower electrode. The 500-nm silicon oxide was thermally-grown dense silicon oxide to improve the stability of the cavity. In addition, a 4-inch SOI with a device layer thickness of 2 μm was prepared, and the top silicon resistivity was P-type with 0.01–0.02 Ω·cm along the crystal orientation. The Buried Oxide Layer was a 100-nm-thick silicon oxide layer. The handle layer was 400-μm-thick P-type silicon with a resistivity of 1–20 Ω·cm and a crystal orientation of <100>. In the first step, the oxide layer of the oxide sheet was masked by a positive 6130 photoresist spun at 3000 rpm, and the thickness of the photoresist after the hard film was 2 μm. A cavity with a depth of 300 nm was formed by reactive ion etching (RIE), and then, the photoresist was removed. So far, oxide sheets with cavities and 2 μm SOI were ready for the next wafer bonding step, as shown in Figure 5a.

#### 2.2.2. Silicon Wafer Bonding

The prepared oxide wafer and SOI with the cavity structure were cleaned with RCA standard. The concentrated sulfuric acid and hydrogen peroxide were used to oxidize and dissolve the metal impurities in the cleaning solution. The ammonia and hydrogen peroxide were used to clean the particles attached to the surface of the silicon wafer and improve its cleanliness, which directly affects the bonding effect. After cleaning and drying, a EVG 510 wafer bonding machine (EVG, St Florian, Austria) was used for the bonding process, which was performed in a vacuum environment at room temperature; the pressure was 200 N, and the process lasted for 3 min. The wafer bonding was successful. At this point, the two wafers are combined by intermolecular forces, and an annealing treatment is required to produce chemical hydrogen bonds between the silicon wafer and SOI, so that the two wafers are closely combined, annealed, and maintained at high temperature to repair any lattice damages.

#### 2.2.3. Micromachining

The upper layer of the bonded silicon wafer, also referred to as the bonded wafer, was composed of a 400-μm handle layer and a 100-nm Buried Oxide Layer, which need to be entirely removed. Firstly, about 300 μm of the handle layer were thinned by a chemo-mechanical polishing (CMP) thinning process, and the residual silicon was wet etched by tetramethylammonium hydroxide (TMAH). This process naturally stops at the Buried Oxide Layer, where the SOI wafer is performed. The process was performed in TMAH solution with a concentration of 5%. The corrosion process was conducted in a circulating water bath with a constant temperature of 80 °C, and the silicon corrosion rate was 2 μm/min. The silicon was completely corroded, the process stopped at the surface of the Buried Oxide Layer, which was cleaned in deionized water. Subsequently, a buffered oxide etch (BOE) solution was prepared to completely corrode the upper and lower surfaces of the oxide layer. The process is easy to perform since silicon is hydrophilic and silicon oxide is hydrophobic. At the end of the process, the silicon surface has undergone obvious changes, which are easy to distinguish.

#### 2.2.4. Isolation Groove Etching, Electrical Isolation, and Metal Graphics

After the oxide layer corrosion process was completed, the isolation groove was etched to improve the independence of the elements. A positive 6130 photoresist spun at 3000 rpm was selected for the mask, and an Omega LPX Dsi (SPTS Technologies Ltd, Newport, UK) deep silicon etching machine (DRIE) was used for etching. The 2-μm-thick silicon film was etched through the mask until it stopped at the oxide layer, so that the isolation groove was etched. In the next step, the electrical isolation layer was prepared, which was deposited in a ICPCVD SI500D (Sentech, Berlin, Germany)plasma enhanced chemical vapor deposition instrument, and a 200-nm-thick silicon oxide layer was deposited on the diaphragm surface under vacuum conditions. After processing, a 500-nm layer of Al was sputtered on the upper and lower surfaces of the silicon wafer by an EXLPLORED magnetron sputtering coating machine. The flow chart is presented in Figure 5. The thick photoresist mask was used for the patterning of the upper electrode, and the positive 4620 photoresist spun at 3000 rpm was used as the mask. After the development film was exposed, wet etching was conducted in phosphoric acid, and the patterning of the upper electrode was completed. In order to achieve good Ohmic contact, the bottom metal underwent an alloy treatment process at 450 °C for 30 min, which was performed in an annealing furnace.

#### 2.2.5. Metal Wire Bonding

**Figure 6:**
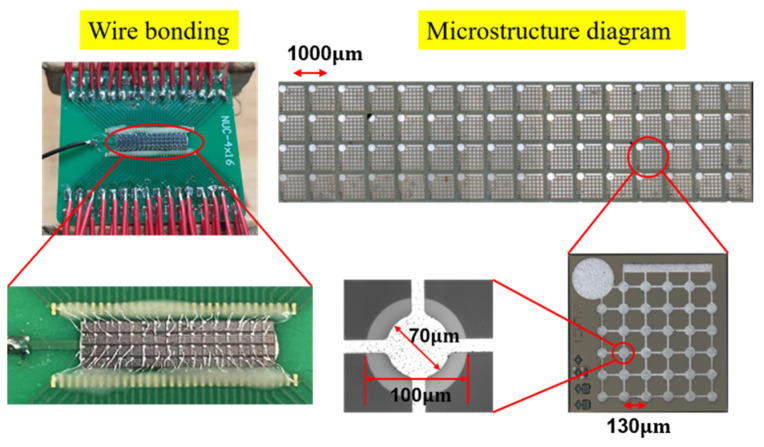
Schematics and images of the resulted transducer. The CMUT array is fixed on the circuit board by Ag–Al wire bonding. The partially enlarged view displays the internal details of the transducer, as well as the stacked structure of the electrode and the cavity.

The prepared transducer was divided into a specified array by slicing, and the CMUT array was adhered to the printed circuit board (PCB). The bottom electrode of the transducer was bonded with a conductive epoxy resin, and the top electrode was connected with the solder joint of the transducer and the circuit board using Ag wire. When the sensor works underwater at the central frequency, the wavelength of the ultrasonic wave is about 370 μm, which is much larger than the diameter of the Ag line of lead bonding. This method prevents the introduction of through holes and simplifies the production steps significantly. The results are presented in Figure 6. Subsequently, the performance of the prepared transducer was characterized.

## 3. Results

### 3.1. Acoustic Test

#### Acoustic Transceiver

**Figure 7:**
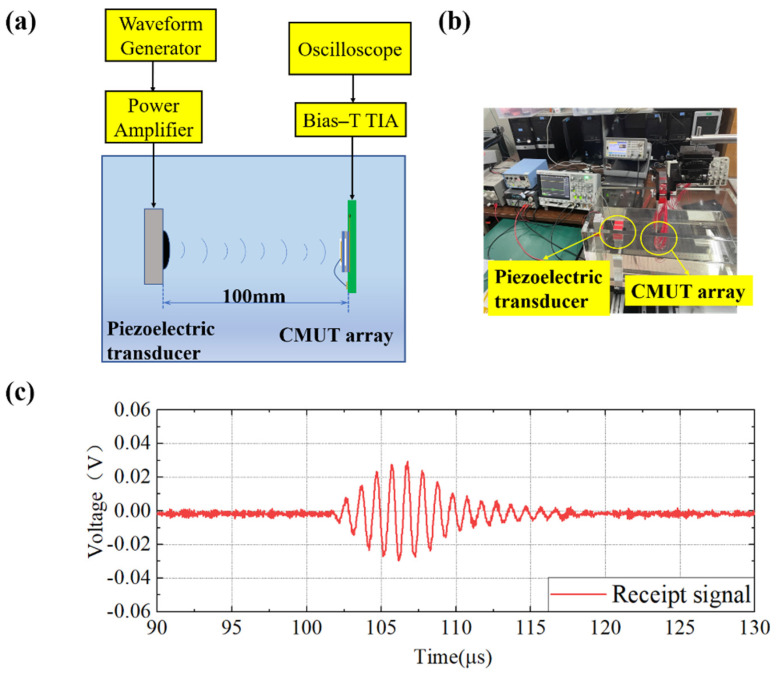
CMUT receiving test. (**a**) Schematic diagram of the CMUT array receiving experiment; (**b**) Photo of the experimental configuration; (**c**) Display of the received signal on the oscilloscope.

First, the receiving characteristics of the prepared transducer were tested. A piezoelectric transducer was selected to emit ultrasound in silicone oil at the same horizontal plane with the CMUT array, which was fixed 100 mm away. The piezoelectric transducer was driven by a waveform generator (Agilent 33521A, Agilent Technologies, Santa Clara, CA, USA) and a power amplifier (NF HSA4101, NF, Yokohama, Japan). The peak-to-peak value of the AC drive voltage was 10 V, and five periodic pulses were emitted. The receiving end was connected to a 25 V DC bias through Bias-T. Then, the received signal was amplified by a transimpedance amplifier (TIA) and displayed on an oscilloscope (KEYSIGHT DSOX3024T, Keysight Technologies, Santa Rosa, CA, USA). The experimental configuration is depicted in Figure 7.

Based on the time difference of the test results, the ultrasonic signal emitted by the transducer at 100 mm was consistent with the theoretical calculation. It can be observed that the received signal exhibited a tailing phenomenon, and the number of signal cycles was not easy to distinguish. According to the above experimental results, the prepared transducer has the function of receiving ultrasonic waves.

**Figure 8:**
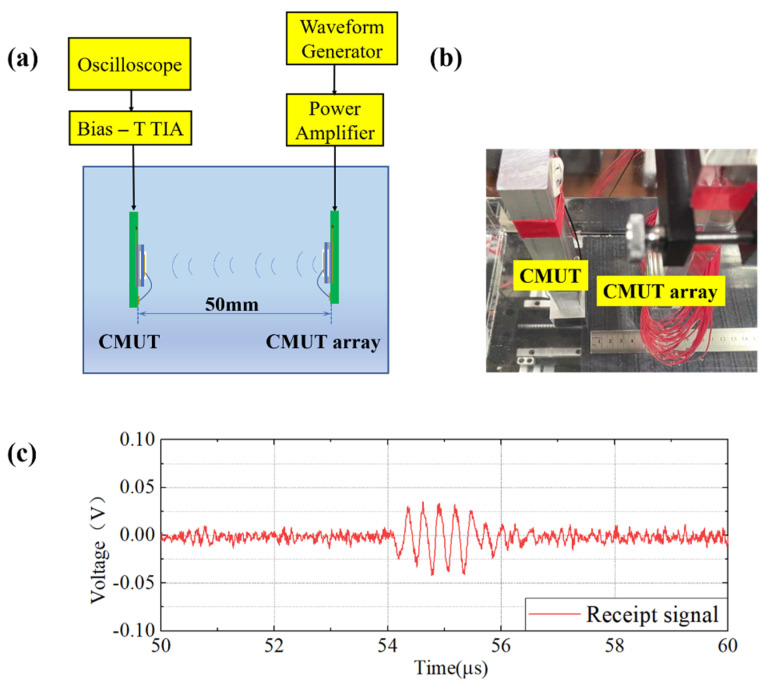
CMUT emission test. (**a**) Schematic diagram of the CMUT array emission experiment; (**b**) Photo of the experimental configuration; (**c**) Display of the received signal on the oscilloscope.

In the emission ultrasound experiment of the CMUT array, the 2D CMUT array was fixed on a mobile platform. The power amplifier used to drive the CMUT array was HSA4101, NF Corporation, Kanagawa, Japan. The power amplifier could replace the Bia-T circuit to couple the AC signal and the DC bias, which was used to directly drive the CMUT. The distance between the CMUT and the prepared CMUT array was 50 mm. The device diagram is presented in Figure 8. Emission and reception took place in the same axial line and were parallel to each other. The driving signal of the CMUT array was a 25 V DC bias voltage and five cycles of AC signal with a peak-to-peak value of 5 V. The experimental device diagram and the experimental results are demonstrated in Figure 8.

After comparing the results, the receiving time of the ultrasonic signal transmitted at a distance of 50 mm is consistent with the theoretical calculation. From the signal perspective, it can be clearly observed that the signal had five cycles, no tailing phenomenon was apparent, while the signal was slightly distorted. Thus far, the prepared CMUT has the function of sending and receiving ultrasonic signals.

### 3.2. Transducer Performance Test

#### 3.2.1. Bandwidth Test

**Figure 9:**
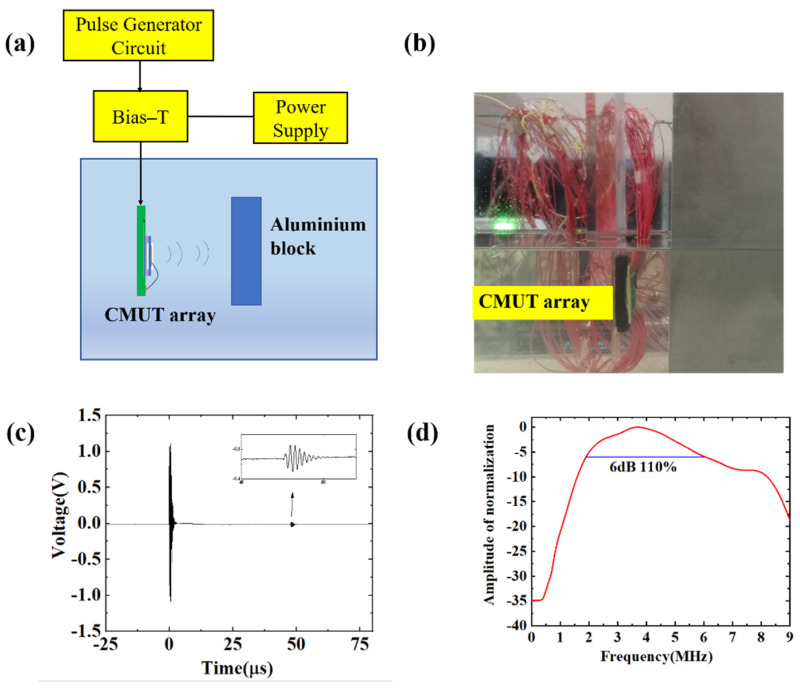
CMUT bandwidth experiment. (**a**) Schematic diagram of bandwidth test principle; (**b**) Photo of the experimental configuration; (**c**) The pulse echo receives the signal; (**d**) Normalized bandwidth curve.

The pulse echo method is used to test the bandwidth. The high voltage pulse excitation is generated by the driving circuit to drive the CMUT to emit ultrasound, which is reflected on the surface of the aluminum block and received by the CMUT again. The received signals are collected, Fourier changes are performed on the signals, and normalized. The bandwidth curve of CMUT is obtained in Figure 9.

In the diagram, it can be observed that the center frequency of CMUT was about 3.8 MHz, the 6 dB bandwidth range was 1.92–6.1 MHz, and the fractional bandwidth reached 110%. This indicates that the prepared transducer has good broadband characteristics.

#### 3.2.2. Receiving Sensitivity Test

**Figure 10:**
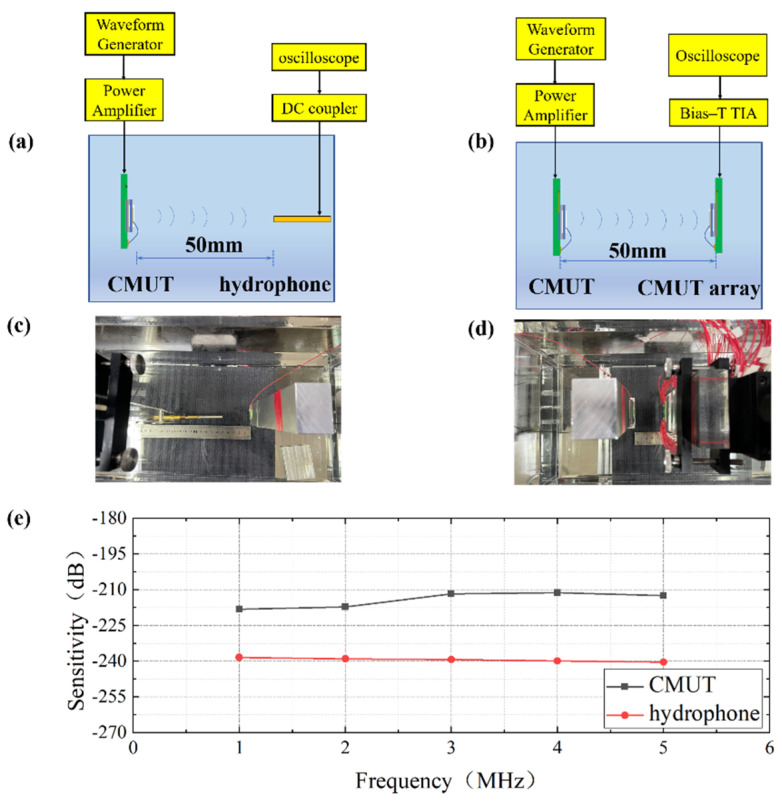
CMUT receiving sensitivity experiment. (**a**) CMUT transmitter with standard needle hydrophone receiver; (**b**) CMUT transmitter with CMUT array receiver; (**c**,**d**) Photos the experimental configurations; (**e**) Sensitivity curves of the CMUT array and the hydrophone.

In this paper, the receiving sensitivity of the CMUT was tested following a comparison method; a schematic and the actual experimental device are presented Figure 10. A CMUT was selected as the transmitting source to transmit ultrasonic waves. The needle hydrophone with known sensitivity received the ultrasonic wave emitted by the transmitting transducer, and the oscilloscope recorded the amplitude of the received signal. Subsequently, the needle hydrophone was replaced by the CMUT array to be tested, and their positions were consistent. The oscilloscope recorded the voltage signal received by a single element of the CMUT array. The ratio of sensitivity was defined as the ratio of signals received in the two experiments. During this process, the transmitting transducer and the signal receiving device were always maintained on the same axis.

Peak-to-peak amplitude of output voltage signal of hydrophone ($V_{H}$), receiving sensitivity of needle hydrophone ($S_{H}$), peak-to-peak amplitude of output voltage signal of CMUT ($V_{C}$), receiving sensitivity of CMUT transducer calculated ($S_{C}$), and receiving sensitivity expressed by decibel value ($S_{CdB}$).

(2) $$S_{C}=\frac{V_{C}}{V_{H}}\times S_{H}\;$$

The base value of 0 dB is 1 V/μPa,

(3) $$S_{CdB}=20logS_{C}-300$$

In the experiment where the receiving sensitivity of the prepared transducer was tested, the driving signal of the transmitting transducer, which was sent by the waveform generator and the power amplifier, was always kept at 20 V DC bias and 5 V AC signal. The DC bias of the transducer was 25 V. The sensitivity was tested at 1 MHz, 2 MHz, 3 MHz, 4 MHz, and 5 MHz. The results indicated that the transducer had better sensitivity than the commercial needle hydrophone.

#### 3.2.3. Conformance Testing

**Figure 11:**
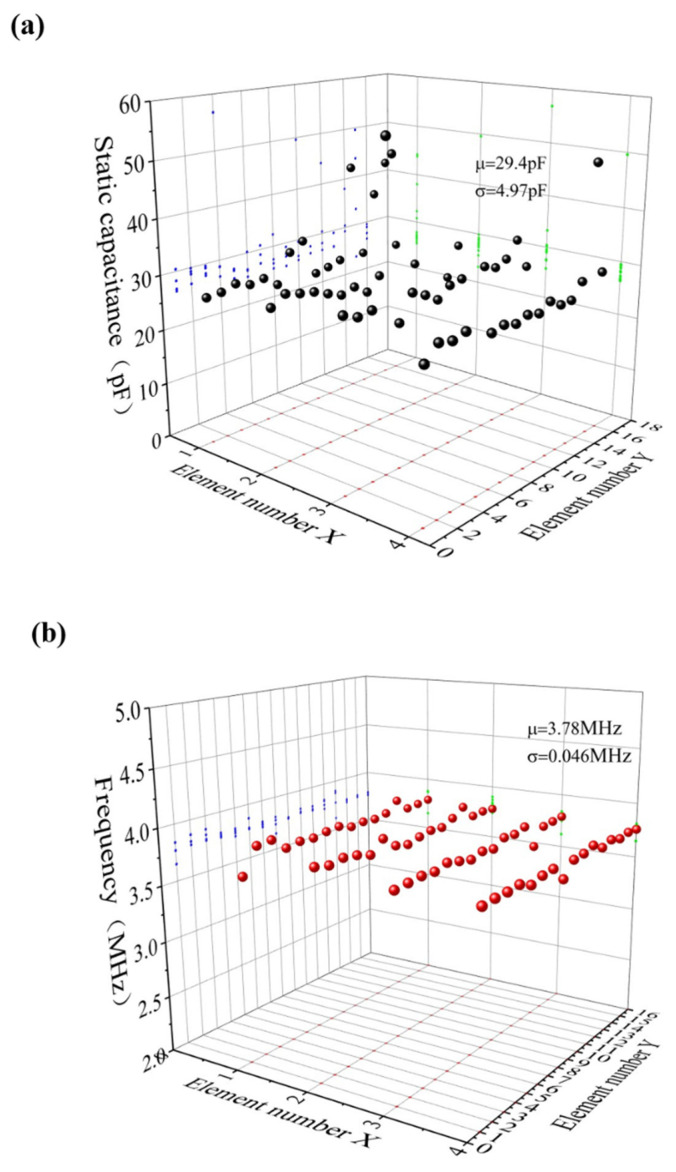
CMUT consistency test results. (**a**) Static capacitance distribution and mean variance of the 64 array elements; (**b**) Resonant frequency distribution and variance of the 64 array elements.

The static capacitance of the 2D array elements at 0 V was measured by an impedance analyzer KEYSIGHT E4990A (Keysight Technologies, Santa Rosa, CA, USA). In total, 64 elements were analyzed. The average capacitance was 29.4 pF, the standard deviation was 4.97 pF, the minimum value was 23.21 pF, the median value was 28.16 pF, and the maximum value was 49.56 pF. The frequency was tested by the scanning method, and the driving signal was 25 V DC bias and 5 V AC signal. According to the results, the average measured resonant frequency was 3.78 MHz and the standard deviation was 46 kHz, which was 1.2% of the average resonant frequency. Figure 11 shows the functional 3D plot between the resonant frequency of the CMUT array and the number of components. It is concluded that the 2D array has good consistency.

### 3.3. Underwater Imaging Experiment

**Figure 12:**
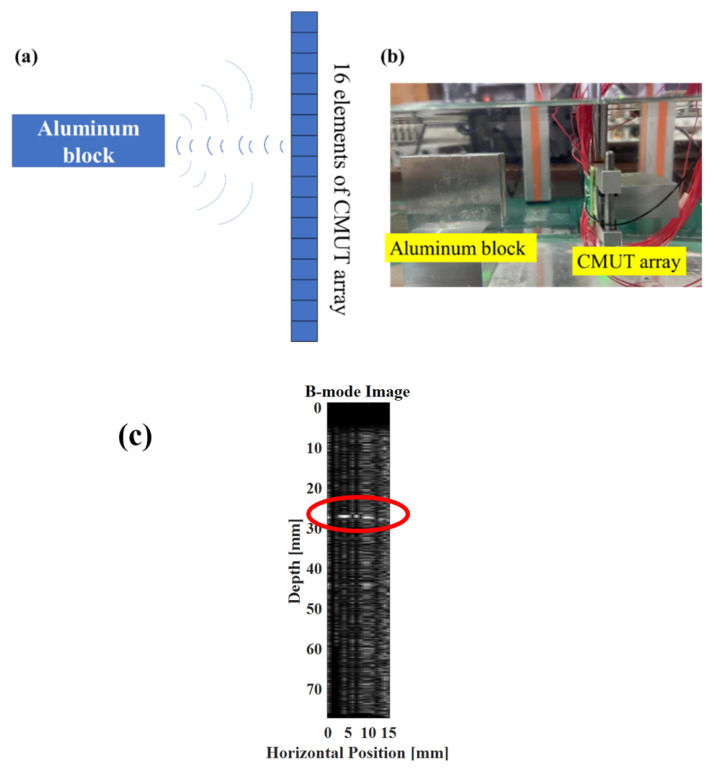
CMUT emission array imaging experiment. (**a**) Schematic diagram of CMUT single line array imaging; (**b**) Photo of the experimental configuration; (**c**) Imaging result: the red circle indicates the first reflective surface of the aluminum block.

In order to verify the array characteristics of the transducer, a preliminary imaging experiment of the obstacle was performed in silicone oil using the 16 elements of the array. The selected obstacle was an aluminum block with a thickness of 10 mm, which was 28 mm away from the transducer, and was aligned with the transducer array. A waveform generator and a power amplifier were combined to drive the CMUT array, the CMUT was driven by sinusoidal pulse signal with period number of 5, peak-to-peak value of 20 V and DC bias voltage of 80 V. One element of the CMUT array emitted ultrasonic waves, and the other 15 transducers were used to receive the ultrasonic waves reflected on the surface of the aluminum block. After the ultrasonic wave emitted by the transducer reached the surface of the aluminum block, it was diffused. The ultrasonic signal reflected in the direction of the transducer was detected. Based on the delay information of the detection signal, the surface of the aluminum block was visualized, and the obtained image is shown in Figure 12. The interface position of the aluminum block is roughly observed in the figure, and the distance between aluminum block and transducer was also approximately 28 mm, which preliminarily verifies the applicability of the array in imaging.

## 4. Discussion

This study investigated the application potential of a CMUT array in underwater imaging, and discussed its prospects from the aspects of device structure and manufacturing. A 2D array with an array element isolation slot was fabricated by the MEMS manufacturing technology. The operating frequency was 3.8 MHz, which meets the requirements of underwater imaging applications. The emission, reception, and bandwidth characteristics of the CMUT array were assessed in silicone oil. The results suggest that the 2D CMUT array fabricated following the newly proposed process meets the basic requirements of underwater imaging. Nevertheless, in order to meet the requirements of 3D ultrasound imaging, the transducer needs to be further optimized, and the transmitting and receiving capabilities must be improved.

The designed CMUT element has multiple cells, which can enhance the emission and reception performances. There are some other issues to watch out for: the resistance effect of the substrate silicon can be equivalent to the series resistance of the capacitor, which does cause negative effects after experimental tests. In the experiment, we found different degrees of attenuation on the static capacitance of existing CMUT by connecting resistors of different resistance values in series. At present, the resistivity is 0.0015 Ω·cm. In order to reduce the influence of parasitic resistance, the next step is to try to use substrate silicon with lower resistivity or develop some compensation measures on the circuit. The delay focusing performance of the array was not investigated in this work, and it will be further analyzed in our future research.

It should be noted that the proposed 2D CMUT array has great research significance in a wide range of medical imaging applications, such as breast ultrasound imaging and cardiac blood flow imaging. The realization of underwater 3D imaging depends on the 2D CMUT array, which, based on the results of this study, can play an important role in real-time 3D ultrasound imaging.

## 5. Conclusions

In this paper, a 2D CMUT array based on silicon material was successfully fabricated following a silicon wafer bonding process. The CMUT working frequency is 3.8 MHz and the 6 dB bandwidth is 110%, which make it suitable for underwater ultrasonic imaging. Compared to silicon nitride thin film, the frequency is easy to control. A silicon oxide electrical isolation layer is introduced between the upper electrode and the diaphragm, eliminating the capacitance coupling. In the process flow, the introduction of through holes is avoided, simplifying the processing steps. The device tests revealed that the sensitivity of the transducer display was significantly improved compared with that of the market standard needle hydrophone. Finally, the linear array was used to image an obstacle in silicone oil, and a preliminary obstacle boundary graph visualization was successfully achieved, which lays the hardware foundation and preliminary experimental research for the later use of 2D arrays in 3D ultrasound imaging applications.

## Figures and Tables

**Table 1:** CMUT array parameters and dimensions.

Parameter	Description/Dimension
Membrane shape	Round
Membrane diameter size	100 µm
Membrane thickness	2 µm
Cavity height	0.3 µm
Electrode thickness	0.5 µm
Trench width	60 µm
Top electrode insulation layer thickness	0.2 µm
Number of cells	35 µm
Transducer dimensions	1000 × 1000 µm

## Data Availability

Not applicable.
